# Cranberry Ingestion Modulated Drug Transporters and Metabolizing Enzymes: Gefitinib Used as a Probe Substrate in Rats

**DOI:** 10.3390/molecules27185772

**Published:** 2022-09-06

**Authors:** Chung-Ping Yu, Pei-Ling Tsai, Pei-Ying Li, Pei-Wen Hsu, Shiuan-Pey Lin, Pei-Dawn Lee Chao, Yu-Chi Hou

**Affiliations:** 1 School of Pharmacy, College of Pharmacy, China Medical University, Taichung 406040, Taiwan; 2 Department of Pharmacy, China Medical University Hospital, Taichung 404332, Taiwan

**Keywords:** cranberry, gefitinib, drug transporter, cytochrome P450s, pharmacokinetics

## Abstract

Cranberry, a polyphenol-rich functional food, is commonly used for the prophylaxis of urinary tract infections. Gefitinib, an anticancer agent clinically prescribed to treat non-small-cell lung cancer, is a substrate of P-glycoprotein (P-gp) and breast cancer resistance protein (BCRP), and metabolized mainly by cytochrome P450 (CYP) 3A4 and CYP2D6. This study used gefitinib as a probe substrate to investigate the modulation of cranberry on P-gp, BCRP, CYP3A4 and CYP2D6. Rats were administered gefitinib with and without 5.0 g/kg of cranberry as juice (CJ). The concentration of gefitinib in serum was determined by LC-MS/MS. The results showed that CJ significantly increased the C_max_ and AUC_0-t_ of gefitinib by 28% and 55%, respectively. Mechanism studies indicated that CJ activated P-gp, and cranberry metabolites (CM) inhibited CYP2D6. Moreover, the protein level of P-gp in rat enterocytes was decreased, whereas that in hepatocytes was increased. In addition, the protein levels of BCRP, CYP3A4 and CYP2D6 in enterocytes and hepatocytes were decreased. In conclusion, CJ ingestion affected the activities and protein levels of P-gp, BCRP, CYP3A4 and CYP2D6.

## 1. Introduction

Cranberry (fruit of *Vaccinium microcarpon*) is distributed in North America and has been popularly used as functional food for the prevention of urinary tract infections [1,2]. In addition, cranberry has shown several beneficial bioactivities, such as anticancer [3], chemoprevention [4] and cardioprotection effects [5,6]. The chemical constituents of cranberry include various polyphenols, such as proanthocyanidins, anthocyanins, flavonoids and phenolic acids [1,7].

Gefitinib, a selective epidermal growth factor receptor tyrosine kinase inhibitor (EGFR-TKI), is clinically used for the treatment of locally advanced or metastatic non-small-cell lung cancer [8]. In the pharmacokinetic aspect, the absorption of gefitinib was moderately slow [9], the bioavailability was 57–59% and the elimination half-life was longer than 24 h [10]. The transport of gefitinib was mediated by P-glycoprotein (P-gp) and breast cancer resistance protein (BCRP) [8,11], and its metabolism was mainly catalyzed by cytochrome P450 (CYP) 3A4 and CYP2D6 [8,10]. The excretion of gefitinib was predominantly via feces, with less than 7% via the renal route [8]. In this study, gefitinib was employed as a probe substrate of P-gp, BCRP, CYP3A4 and CYP2D6, and the effect of CJ ingestion on the pharmacokinetics of gefitinib was investigated in rats.

P-gp and BCRP are the major efflux transporters of xenobiotics [12,13,14]. CYP 3A4 and CYP2D6 are the most important CYP isoenzymes responsible for the metabolism of more than 50% and 25% of drugs, respectively [15,16,17]. Cranberry polyphenols have shown inhibitions on the activities of P-gp, BCRP, CYP3A4 and CYP2D6 [13,18,19], based mostly on in vitro studies evaluating the parent form of polyphenols. However, nowadays it has been well recognized that most polyphenols undergo rapid and extensive metabolism after absorption, and the major forms in the body are the conjugated metabolites instead of the parent forms [20,21]. Therefore, this study in turn used cranberry metabolites (CM) to evaluate the modulation effect of CJ on CYP2D6. Furthermore, the protein levels of P-gp, BCRP, CYP3A4 and CYP2D6 in the enterocytes and hepatocytes of rats were determined after CJ ingestion.

## 2. Results

### 2.1. Quantitation of Gefitinib and O-Desmethyl Gefitinib in Serum

**Figure 1:**
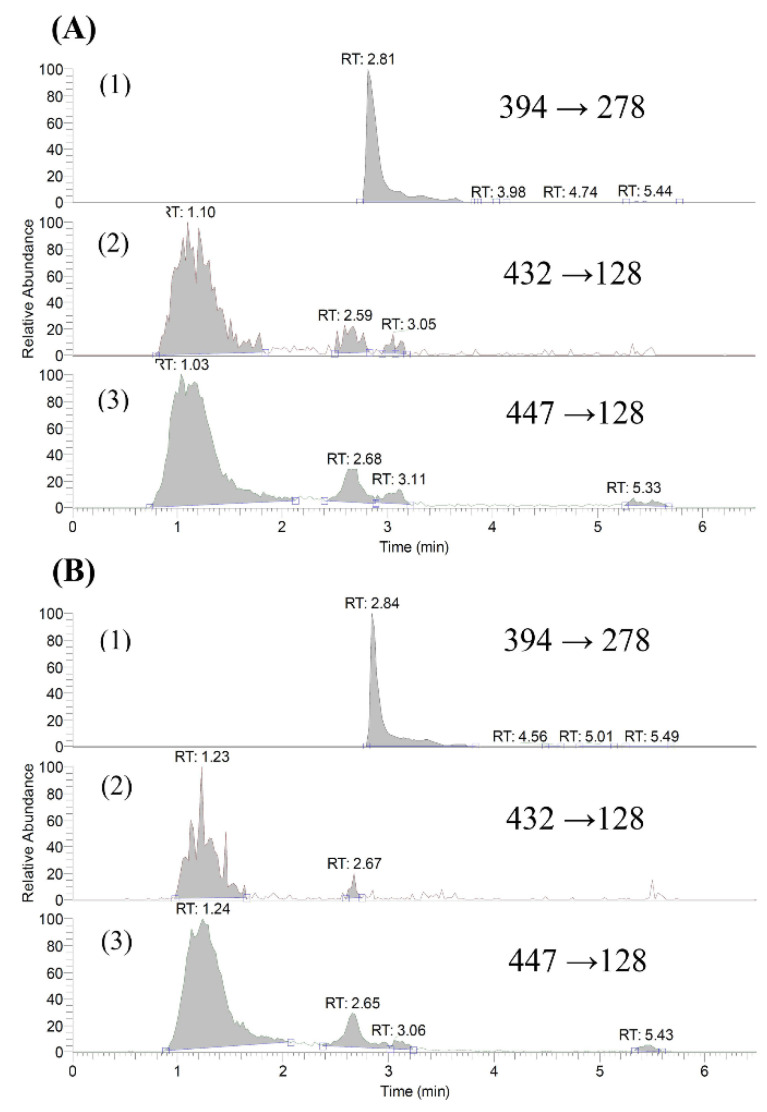
LC-MS/MS chromatograms: (**A**) blank serum spiked with erlotinib (1, internal standard), *O*-desmethyl gefitinib (2) and gefitinib (3); (**B**) a serum sample obtained at 240 min after a dose of gefitinib.

An LC-MS/MS method using a mixture of acetonitrile and 0.1% formic acid as the mobile phase was developed and validated for the assay of gefitinib and O-desmethyl gefitinib in serum. Figure 1 shows the LC-MS/MS chromatograms of gefitinib and O-desmethyl gefitinib in blank serum and a serum sample. The calibration of gefitinib and O-desmethyl gefitinib in the ranges of 5.0–0.08 and 0.50–0.03 μg/mL showed good linearities, respectively. However, the concentrations of O-desmethyl gefitinib were below LLOQ in most serum samples.

Validation of the analytical method for gefitinib showed that coefficients of variation and the relative errors were below 13.0% and 14.0%, respectively. The recoveries of gefitinib from serum were 88%, 90% and 104% at concentrations of 0.13, 0.25 and 0.50 μg/mL, respectively. These results indicated that the precision, accuracy and recovery of this analytical method were satisfactory, and all met the criteria of the Guidance on Bioanalytical Method Validation from the FDA (May 2018).

### 2.2. Effect of CJ Intake on Gefitinib Pharmacokinetics in Rats

**Figure 2:**
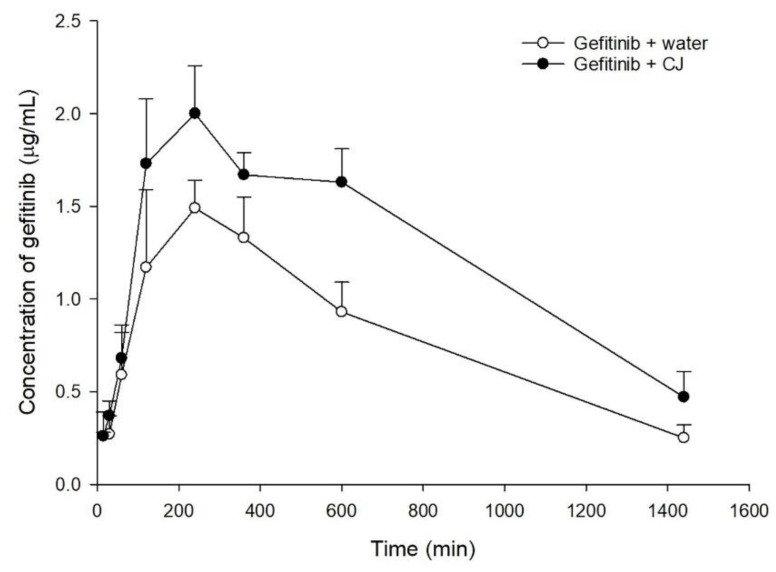
Mean (±S.E.) serum concentration–time profiles of gefitinib after oral administration of gefitinib alone (○, 50 mg/kg) and coadministration with CJ (●, 5.0 g/kg of cranberry) in rats.

The serum gefitinib concentration–time profiles after oral administration of gefitinib alone and coadministration with 5.0 g/kg of cranberry are shown in Figure 2. The pharmacokinetic parameters of the two treatments are listed in Table 1. When gefitinib was coadministered with cranberry, the C_max_ and AUC_0-t_ of gefitinib were significantly enhanced by 28% and 55%, respectively.

### 2.3. Effect of CJ on P-gp Activity

**Figure 3:**
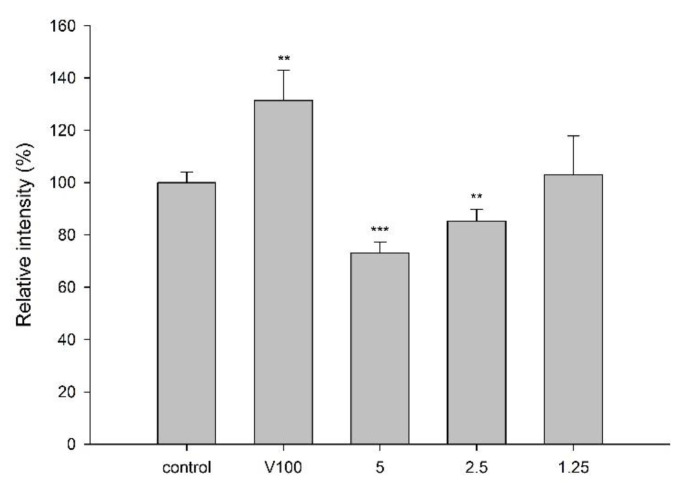
Effect of CJ (mg/mL) on the intracellular accumulation of R123 in LS 180 cells. V: verapamil (100 μM, positive control of P-gp inhibitor). ** p < 0.01 and *** p < 0.001.

LS 180 cell line was used for the transport assay of rhodamine 123 (R123), a fluorescent substrate of P-gp, to evaluate the effect of CJ on P-gp activity. MTT assay showed that incubation of LS 180 with 5.0 mg/mL of cranberry for 24 h exhibited no influence on the cell viability. The intracellular accumulation of R123 in the presence and absence of CJ is shown in Figure 3, indicating that CJ significantly decreased the intracellular accumulation of R123 by 27%. On the contrary, verapamil (100 μM), a positive control of P-gp inhibitor, significantly increased the intracellular accumulation of R123 by 31%.

### 2.4. Effect of CM on CYP2D6 Activity

**Figure 4:**
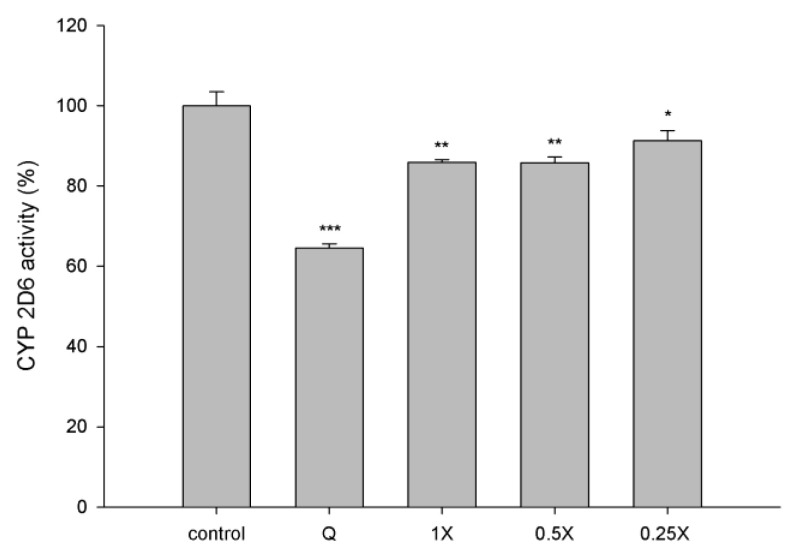
Effects of CM (1-, 0.5- and 0.25-fold serum concentration) on the activity of CYP2D6. Q: quinidine (10 μM in blank serum, positive control of CYP2D6 inhibitor). * p < 0.05, ** p < 0.01 and *** p < 0.001.

The effect of CM on CYP2D6 activity is shown in Figure 4. CM at 1-, 0.5- and 0.25- fold serum concentrations significantly decreased CYP2D6 activity by 15%, 14% and 7%, respectively, when compared to those of correspondent concentration of blank serum specimens. As a positive control of CYP2D6 inhibitor, quinidine (10 μM in blank serum) significantly decreased the CYP2D6 activity by 36%.

### 2.5. Effect of CJ Ingestion on the Protein Levels of P-gp, BCRP, CYP3A4 and CYP2D6

**Figure 5:**
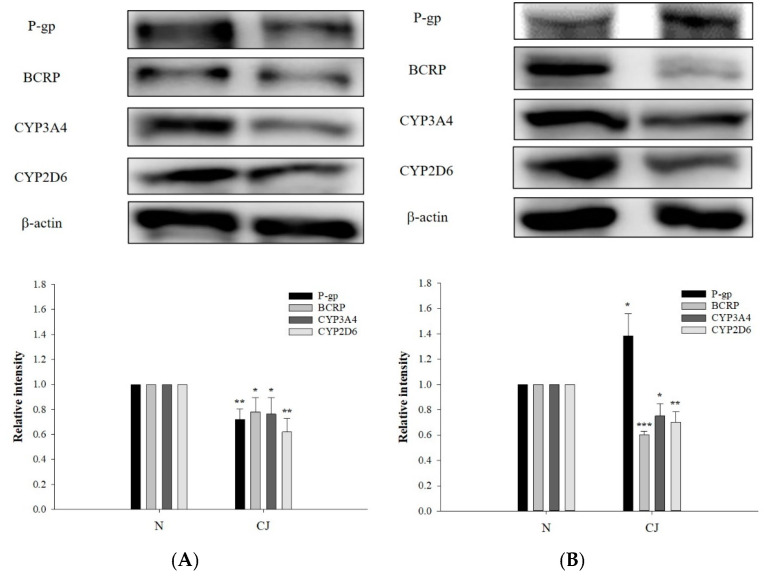
Protein levels (mean ±S.D.) of P-gp, BCRP, CYP3A4 and CYP2D6 in enterocytes (**A**) and hepatocytes (**B**) after ingestion of water (N, n = 3) and CJ (5.0 g/kg) (n = 3). * p < 0.05, ** p < 0.01 and *** p < 0.001.

The protein levels of P-gp, BCRP, CYP3A4 and CYP2D6 in rat enterocytes and hepatocytes after CJ ingestion are shown in Figure 5, indicating that CJ ingestion significantly decreased the protein level of P-gp by 28% in enterocytes, whereas that in hepatocytes was significantly increased by 39%. The protein levels of BCRP were significantly reduced in enterocytes and hepatocytes by 22% and 40%, respectively. The protein levels of CYP3A4 were significantly lowered by 24% and 25%, while those of CYP2D6 were significantly decreased by 38% and 30%, respectively. These results indicated that CJ downregulated the protein levels of BCRP, CYP3A4 and CYP2D6 in both organs, whereas P-gp protein was downregulated in enterocytes and upregulated in hepatocytes.

## 3. Discussion

The quantitation method of gefitinib and O-desmethyl gefitinib in serum using LC-MS/MS was developed in this study. Validation of this method indicated that the precision, accuracy and recoveries were satisfactory. Therefore, this analytical method was suitable for the pharmacokinetic study of gefitinib and its metabolite O-desmethyl gefitinib. However, the concentrations of O-desmethyl gefitinib in all serum samples were below the LLOQ and could not be quantitated in this study.

Gefitinib was used as a probe substrate of P-gp, BCRP, CYP3A4 and CYP2D6 in rats to observe the pharmacokinetic influence of CJ. The rat model has been used in the preclinical studies of gefitinib, because rats have orthologous genes of P-gp, BCRP, CYP3A4 and CYP2D6 to humans [22,23]. The results of the pharmacokinetic study showed that coadministration of CJ increased the peak serum concentration and the systemic exposure of gefitinib, indicating that the oral bioavailability of gefitinib was enhanced by CJ. Observation of the serum profiles found that two curves within 2 h were essentially superposable, implying that the absorption phase of gefitinib was not affected by CJ. Accordingly, we could infer that CJ ingestion increased the bioavailability of gefitinib during the elimination phase, and we then proceeded to verify whether the metabolism and/or excretion of gefitinib were hampered after CJ ingestion by employing in vitro, ex vivo and in vivo models.

Concerning the involved mechanisms, the modulation effect of CJ on drug transporters P-gp and BCRP, and metabolizing enzymes CYP3A4 and CYP2D6 were evaluated. For the assessment of P-gp activity, an in vitro transport study of R123 was conducted. For the evaluation of CYP2D6 activity, an ex vivo study using CM mimicking the virtual metabolites in the body was performed. For determining the influences on protein levels of P-gp, BCRP, CYP3A4 and CYP2D6, an in vivo study using two groups of rats individually administered with CJ and water was carried out. Then, the specific proteins in the enterocytes and hepatocytes were analyzed and compared between the two groups.

The result of the transport study of R123 showed that CJ decreased the intracellular accumulation of R123, indicating that CJ increased the activity of P-gp. However, the protein level of P-gp in the enterocytes was decreased, which might reverse the effect of P-gp activation. Conversely, the protein level of P-gp in the hepatocytes was increased. Therefore, we considered that these opposite influences on P-gp protein level in the enterocytes and hepatocytes might cancel out each other and result in negligible influence on the absorption of gefitinib. Taken together, the modulations on P-gp, including activity and protein level, might not lead to significant pharmacokinetic alteration of gefitinib.

Besides P-gp, the activity and protein level of BCRP might play roles in the mechanisms of CJ–gefitinib interaction. Recently, a previous study of cranberry–warfarin interaction reported that CJ activated BCRP, whereas CM inhibited BCRP [24], implying that these effects might cancel out each other in vivo. Furthermore, the results of protein analysis showed that BCRP protein in enterocytes and hepatocytes was decreased, indicating that the excretion of gefitinib to feces via BCRP might be reduced. Therefore, both the inhibition of BCRP activity caused by CM and the decreased protein level of BCRP after CJ ingestion could explain the decreased excretion of gefitinib via feces.

Regarding the modulation of CYPs, the results showed that CM inhibited the activity of CYP2D6. By referring to a previous report [24], CM inhibited the activities of both CYP3A4 and CYP2D6, two major metabolic enzymes of gefitinib. Furthermore, the results of protein analysis indicated that CJ ingestion decreased the protein levels of CYP3A4 and CYP2D6 in the enterocytes and hepatocytes of rats, suggesting that CJ ingestion might reduce the metabolism of gefitinib. To sum up, the activities and protein levels of CYP3A4 and CYP2D6 were decreased after CJ ingestion, which could account for the decreased elimination of gefitinib.

Based on the above evidence, CJ ingestion decreased the protein expressions of BCRP, CYP3A4 and CYP2D6; moreover, the activities of BCRP, CYP3A4 and CYP2D6 were inhibited. Therefore, we suspect that CJ ingestion might decrease the excretion of substrate drugs of BCRP, such as topotecan, irinotecan, imatinib, sunitinib, methotrexate, abacavir, sulfasalazine and cimetidine [25,26]. On other hand, the metabolism of drugs catalyzed mainly by CYP3A4 and CYP2D6, such as erlotinib, etoposide, paclitaxel, everolimus, tacrolimus, indinavir, tamoxifen, haloperidol and amitriptyline, might be inhibited after CJ ingestion [27,28,29,30]. Therefore, cranberry has a potential risk to increase the systemic exposure of substrate drugs of BCRP, CYP3A4 and CYP2D6. We suggested that cranberry ingestion is better avoided for patients treated with the above-mentioned critical medicines.

## 4. Materials and Methods

### 4.1. Chemicals and Reagents

Gefitinib (purity 98%) and erlotinib (purity 98%) were purchased from Wuhan Sunrise Technology Development, Inc. (Wujiashan, China). *O*-Desmethyl gefitinib (purity 98%) was obtained from Toronto Research Chemicals Inc. (North York, ON, Canada). R123, dimethyl sulfoxide (DMSO), sodium dodecyl sulfate (SDS), quinidine (purity 98%), 3-(4′,5′-dimethylthiazol-2′-yl)-2,5-diphenyltetrazolium bromide (MTT), Triton X-100 and formic acid were provided by Sigma (St. Louis, MO, USA). Acetonitrile, ethyl acetate and methanol with LC-MS grade were obtained from J.T. Baker Inc. (Center Valley, PA, USA). Fetal bovine serum (FBS) was obtained from Biological Industries Inc. (Kibbutz, Beit Haemek, Israel). Dulbecco’s modified Eagle medium (DMEM), Hank’s buffered salt solution (HBSS), 4-(2-hydroxyethyl)-1-piperazineethanesulfonic acid (HEPES), nonessential amino acid (NEAA) and trypsin/EDTA were purchased from Invitrogen (Grand Island, NY, USA). Antibodies against ABCB1 and ABCG2 were supplied by Abcam (Cambridge, UK). Antibodies against CYP3A4, CYP2D6 and β-actin were purchased from GeneTex (San Antonio, TX, USA). Polyvinylidene fluoride transfer membranes (Immobilon P) and chemiluminescence (ECL) were obtained from Millipore Corp. (Millipore, Bedford, MA, USA). Milli-Q plus water (Millipore, Bedford, MA, USA) was used for all processes.

### 4.2. Characterization of Polyphenols in CJ

The preparation and characterization of CJ (0.5 g/mL of cranberry) were reported in our previous study [24]. Briefly, a total of 1.5 kg of cranberries was blended with 1.5 L of water. Then, sufficient water was added to make 3 L to afford 0.5 g/mL of CJ. Three hundred microliters of CJ was mixed with 700 μL of methanol. After centrifugation, the supernatant was mixed with an equal volume of internal standard solution (0.01 μg/mL of 6,7-dimethoxycoumarin in methanol), and 5 μL was subject to LC-MS/MS analysis, which was performed on a high-performance liquid chromatographic (HPLC) system equipped with Accela 1250 pump and auto-sampler (Thermo Fisher Scientific Inc., Waltham, MA, USA). Chromatographic separation of analytes was achieved using a Thermo Hypersil GOLD C18 analytical column (50 mm × 2.1 mm, 1.9 μm) with a prefilter. The mobile phase consisted of 0.01% formic acid (A) and acetonitrile containing 0.01% formic acid (B), and a gradient elution was programmed as follows: A/B: 80/20 (0–1.0 min), 20/80 (1.1–4 min) and 80/20 (4.1–8.0 min). The flow rate was 0.2 mL/min. The column effluent was detected by H-ESI (heated-electrospray ionization)-II probe with Quantum Access MAX triple stage quadrupole (TSQ) mass spectrometer (mass range: 10 to 3000 *m/z*) (Thermo Fisher Scientific Inc., USA).

### 4.3. Animals, Drug Administration and Blood Collection

All animal experiments adhered to “The Guidebook for the Care and Use of Laboratory Animals” published by the Chinese Society for the Laboratory Animal Science, Taiwan. The protocol was approved by the Institutional Animal Care and Use Committee of China Medical University (Taichung, Taiwan) (CMUIACUC-2017-096). The anesthesia was performed under 2–3% isoflurane, and all efforts were made to minimize the suffering of rats.

Twelve male Sprague-Dawley rats (300–400 g) were supplied by BioLASCO Taiwan Co., Ltd. (Yilan, Taiwan) and randomly divided into three groups (six rats in each group). Gefitinib solution was prepared by mixing 300 mg of gefitinib with 1.5 g of malic acid and dissolved in 15 mL of deionized water to afford a concentration of 20 mg/mL. Rats were fasted for 12 h before dosing, and food was offered 3 h after drug administration. In the 1st group, rats were orally given gefitinib (50 mg/kg) with water (10 mL/kg) via gastric gavage. In the 2nd group, rats were orally given gefitinib (50 mg/kg) with CJ (5.0 g/kg). The blood samples of rats were withdrawn at 15, 30, 60, 120, 240, 360, 600 and 1440 min after administration of gefitinib. The serum was collected after centrifugation at 10,000× *g* for 15 min and stored at −20 °C until analysis.

### 4.4. Quantitation of Gefitinib and O-Desmethyl Gefitinib in Serum and Method Validation

The serum concentrations of gefitinib and O-desmethyl gefitinib were quantitated by LC-MS/MS. Briefly, 50 μL of serum was partitioned with an equal volume of ethyl acetate containing erlotinib (0.5 μg/mL) as internal standard. The ethyl acetate layer was evaporated under N_2_ gas to dryness and reconstituted with 50 μL of acetonitrile containing 0.1% formic acid, and then 5 μL was subject to LC-MS/MS analysis.

The mobile phase consisted of 0.1% formic acid (A) and acetonitrile containing 0.1% formic acid (B), and a gradient elution was programmed as follows: A/B: 80/20 (0–1.0 min), 0/100 (1.0–3.5 min) and 80/20 (3.5–6.5 min). The flow rate was 0.2 mL/ min. Nitrogen was used as sheath gas at 50 arbitrary units and auxiliary gas at 10 arbitrary units. The collision energy was set at 30 eV, spray voltage at 3000 V, capillary temperature at 203 °C, vaporizer temperature at 192 °C and tube lens offset at 182 V. The following mass transitions were used for SRM analysis: gefitinib (*m/z* 447 → *m/z* 128), O-desmethyl gefitinib (m*/z* 432 → *m/z* 128) and erlotinib (*m/z* 394 → *m/z* 278). ESI-MS spectra were recorded in the positive ion mode.

The precision and accuracy of the analytical method was evaluated by intraday and interday analysis of triplicate standards within one day and over a period of three days. Recoveries of gefitinib from serum were evaluated at concentrations of 0.13, 0.25 and 0.50 μg/mL.

### 4.5. Cell Line and Culture Conditions

LS 180 cell line, intestinal human colon adenocarcinoma, was obtained from the Bioresource Collection and Research Center (Hsinchu, Taiwan). Cells were routinely grown in DMEM containing 10% FBS, 1% NEAA, 1% PSG (containing 100 units/mL of penicillin, 100 μg/mL of streptomycin and 292 μg/mL of glutamine) at 37 °C in a humidified incubator containing 5% CO_2_.

Madin–Darby canine kidney type II cells (MDCK II), transfected with BCRP (MDCK II-BCRP), were kindly provided by Prof. Dr. Piet Borst (Netherlands Cancer Institute, Amsterdam, Netherlands). Cells were grown in DMEM medium supplemented with 10% FBS and 1% PSG at 37 °C in a humidified incubator containing 5% CO_2_.

The medium was changed every other day and cells were subcultured when 80% to 90% confluency was reached.

### 4.6. Cell Viability Assay

The effects of tested agents on the viability of LS 180 cells were evaluated by MTT assay [31]. Cells were seeded in 96-well plates in DMEM culture and allowed to attach overnight. A series of concentrations of tested agents were added to the wells and incubated for 24 h; then 15 μL of MTT (5 mg/mL) was added to each well, and the cells were incubated for a further 4 h. During this period, MTT was reduced to formazan crystal by live cells. Acid-sodium dodecyl sulfate (SDS) (10%) solution was added to solubilize the purple crystal at the end of incubation, and the absorbance was recorded at 570 nm by a microplate reader (Thermo Fisher Scientific Inc., USA).

### 4.7. Effect of CJ on P-gp Activity

LS 180 cell line was used to evaluate the modulation effect of CJ on P-gp. The activity of P-gp was determined by measuring the intracellular accumulation of R123, a fluorescent P-gp substrate, in LS 180 cells [32,33,34]. Briefly, the cells in 96-well (1 × 10^5^ cells/well) plates were incubated with DMEM at 37 °C overnight and then washed three times with ice-cold HBSS. The cells were then incubated with 100 μL of R123 (10 μM) for 1 h. After washing thrice with ice-cold HBSS, a series of concentrations of cranberry were added to each well and incubated for 4 h. HBSS was added as blank control, and verapamil was used as a positive control. The cells were then washed thrice with ice-cold HBSS and lysed with lysis buffer (0.1% Triton X-100). R123 fluorescence was measured with excitation at 485 nm and emission at 528 nm. To quantitate the content of protein in each well, 10 μL of cell lysate was added to 200 μL of protein assay reagent (Bio-Rad, Hercules, CA, USA), and the optical density was measured at 570 nm. The relative intracellular accumulation of R123 was calculated by comparing the fluorescence intensity with that of control after protein correction.

### 4.8. Preparation of CM

In order to mimic the molecules interacting with CYP2D6 in enterocytes and hepatocytes, CM was prepared from rats. The method of preparation was described in our previous study [24]. Briefly, rats were orally given cranberry juice (5.0 g/kg) after overnight fast, and blood was collected 15 min later. The serum obtained after centrifugation was vortexed with 4-fold methanol. After centrifugation at 10,000× *g* for 15 min at 4 °C, the supernatant was concentrated in a rotary evaporator under vacuum to dryness. To the residue, an appropriate volume of water was added to afford a solution with a 10-fold serum concentration, which was divided into aliquots and stored at −80 °C for later use. On the other hand, blank serum was withdrawn from rats and processed likewise to prepare controls for the comparison with correspondent concentrations of CM tested.

### 4.9. Effect of CM on CYP2D6 Activity

To evaluate the modulation effect of CJ ingestion on the activity of CYP2D6, which is one of the metabolizing enzymes of gefitinib, an ex vivo approach employing CM to mimic the molecules interacting with CYP2D6 was conducted. The procedures followed the manual of the Vivid^®^ CYP 450 screening kit (Invitrogen, CA, USA). Briefly, after incubating CM (1-, 0.5- and 0.25-fold serum concentration) and correspondent concentration of blank serum specimen with CYP 450 recombinant BACULOSOMES^®^, glucose-6-phosphate and glucose-6-phosphate dehydrogenase in 96-well black plate at room temperature for 20 min, specific substrate (Vivid^®^ MOBFC for CYP2D6) and NADP^+^ were added and incubated at room temperature for another 30 min. At the end of incubation, quinidine was added to stop the reaction of CYP2D6, and the fluorescence was measured with excitation at 415 nm and emission at 520 nm.

### 4.10. Western Blot Analysis of P-gp, BCRP, CYP3A4 and CYP2D6 in Rat Enterocytes and Hepatocytes

After administration of CJ and water individually, rats (n = 3 in each group) were sacrificed by inhaling CO_2_, and then the organs, including ileum and liver, were removed, blotted dry with filter paper and accurately weighed. The tissues were homogenized and lysed with radioimmune precipitation (RIPA) buffer (Merck, Darmstadt, Germany) and collected. Furthermore, the samples were separated by 10% SDS-polyacrylamide gel electrophoresis and then transferred onto polyvinylidenedifluoride membranes. The membranes were blocked at room temperature for 1 min in the blocking buffer (Goal Bio, TW) and then washed 3 times with 0.1% TBST (Tris-buffered saline with 0.1% Tween^®^ 20 detergent). After washing, the blots were incubated with P-gp, BCRP, CYP3A4, CYP2D6 and β-actin primary antibodies, individually, at 4 °C overnight. Then the blots were washed 3 times with 0.1% TBST and reacted with secondary antibodies at room temperature for 1 h. The blots on the membranes were visualized by ECL reagent (Advansta Inc., San Jose, CA, USA) and detected by using Amersham ImageQuant800 (Cytiva Inc., Marlborough, MA, USA). Then, the protein levels were calculated by using Multi Gauge V3.0 software.

### 4.11. Data Analysis

Pharmacokinetic parameters were calculated by the noncompartment model of Phoenix WinNonlin^®^ (version 8.1 Pharsight Corporation, St. Louis, MO, USA). The peak serum concentration (C_m__ax_) was calculated based on experimental measurement. The areas under the serum concentration–time curves (AUC_0-t_) from time zero to last were calculated by the trapezoidal rule. Each pharmacokinetic parameter was compared among various treatment groups using one-way analysis of variance (ANOVA) with Scheffe’s test. Other studies were statistically compared using unpaired Student’s *t*-test. Statistical significance level was set at *p* < 0.05.

## 5. Conclusions

Ingestion of cranberry affected the activities and protein levels of P-gp, BCRP, CYP3A4 and CYP2D6. The decreased activities and protein levels of BCRP, CYP3A4 and CYP2D6 could account for the increased systemic exposure of gefitinib.

## Figures and Tables

**Table 1:** Mean (±S.E.) pharmacokinetic parameters of gefitinib in rats (n = 5 in each group) after giving gefitinib (50 mg/kg) alone and coadministration with CJ (5.0 g/kg of cranberry).

	Treatments	Gefitinib + Water	Gefitinib + CJ
Parameters			
T_max_ (min)		264.0 ± 44.9	216.0 ± 44.9
C_max_ (μg/mL)		1.8 ± 0.3 ^a^	2.3 ± 0.2 ^b^
			(+28%)
AUC_0-t_ (μg⋅min/mL)		1170.5 ± 129.3 ^a^	1818.2 ± 144.8 ^b^
			(+55%)
MRT (min)		511.3 ± 40.1	543.0 ± 29.8

Data are expressed as means ± S.E. Means in a given row without a common superscript differ at *p* < 0.05. (A mean with superscript “a” was significantly different from a mean with superscript “b”). T_max_: time of maximum serum concentration. C_max_: maximum serum concentration. AUC_0-t_: area under the serum concentration–time curve from time zero to last time. MRT: mean residence time.

## Data Availability

The data that support the findings of this study are not available.

## Sample Availability

Samples of the compounds are available from the authors.

## Abbreviations

ACN	acetonitrile
AUC	the area under the serum concentration-time curve
BCRP	breast cancer resistance protein
CJ	cranberry juice
CYP	cytochrome P450
C_max_	peak blood concentration
CM	serum metabolites of cranberry
DMEM	Dulbecco’s modified Eagle medium
DMSO	dimethyl sulfoxide
ECL	chemiluminescence
FBS	fetal bovine serum
HBSS	Hank’s buffered salt solution
HPLC	high-performance liquid chromatography
MDCKII	Madin-Darby canine kidney type II cells
MRT	mean residence time
MTT	3-(4′,5′-dimethylthiazol-2′-yl)-2,5-diphenyltetrazolium bromide
NEAA	nonessential amino acid
P-gp	P-glycoprotein
PSG	penicillin streptomycin and glutamine
R123	rhodamine 123
SDS	sodium dodecyl sulfate
TBST	Tris-buffered saline with 0.1% Tween^®^ 20 detergent
